# Assessing *Anopheles* species collection techniques in a low malaria transmission area: implications for vector surveillance and control

**DOI:** 10.1186/s12936-025-05463-x

**Published:** 2025-07-01

**Authors:** Thabo Mashatola, Power Tshikae, John Govere, Theresa T. Mazarire, Basil Brooke, Givemore Munhenga

**Affiliations:** 1 Wits Research Institute for Malaria, Faculty of Health Sciences, University of the Witwatersrand, Johannesburg, South Africa; 2 Centre for Emerging Zoonotic & Parasitic Diseases, National Institute for Communicable Diseases, a Division of the National Health Laboratory Service, Johannesburg, South Africa; 3 KwaZulu Natal Department of Health, Malaria Control Programme, Jozini, South Africa; 4 Institute for Sustainable Malaria Control, School of Health Systems and Public Health, Faculty of Health Sciences, University of Pretoria, Pretoria, South Africa

**Keywords:** Collection techniques, Species abundance, Species diversity, Entomological indicators

## Abstract

**Background:**

Effective entomological surveillance is crucial for malaria control, especially in low transmission settings. This study aimed to compare the performance of three mosquito collection methods (clay pots, carbon dioxide (CO_2_)-baited tents, and human landing catches (HLC)) for malaria vector surveillance in the low transmission area of Nkomazi, South Africa.

**Methods:**

From March 2019 to March 2020, adult mosquitoes were collected monthly from three different sites over five consecutive nights and mornings. Each collection method was used at each site to capture both outdoor resting and host-seeking mosquitoes. The collected mosquitoes were morphologically identified to the *Anopheles* genus and species, followed by confirmation using molecular PCR assays. The species composition, relative abundance, and diversity were evaluated, and statistical tests, including Kruskal–Wallis and ANOVA, were used to assess differences in abundance and diversity across collection sites and methods. A Generalized Linear Mixed Model was applied to assess the impact of various factors on species abundance.

**Results:**

A total of 1337 *Anopheles* mosquitoes were collected, with 98.5% being females. CO_2_-baited tents yielded the highest number of mosquitoes (57.6%), followed by HLC (39.1%) and clay pots (3.3%). Species composition included 52.4% of the *Anopheles gambiae* complex and 13.6% of the *Anopheles funestus* group. While species richness varied significantly between collection methods, with CO_2_-baited tents showing the highest richness, no significant differences were observed in abundance across sites or methods. The clay pot method was associated with significantly lower species abundance compared to HLC and CO_2_-baited tents. Species abundance fluctuated across months, with February and November showing a higher record. Males were less prevalent than females. Additionally, species abundance was lower in Block C and Vlakbult compared to Block A.

**Conclusions:**

This study highlights the importance of choosing appropriate mosquito collection methods based on specific entomological indicators and transmission dynamics. While CO_2_-baited tents provided the highest species richness, clay pots, despite yielding fewer mosquitoes, are effective for capturing outdoor resting malaria vectors. These findings suggest that a combination of collection methods is essential for inclusive malaria vector surveillance, facilitating tailored strategies for effective malaria control and resource optimization.

**Supplementary Information:**

The online version contains supplementary material available at 10.1186/s12936-025-05463-x.

## Background

Malaria remains one of the most prevalent and lethal infectious diseases globally. The World Health Organization (WHO) estimates that globally there were 263 million malaria cases and 597 000 malaria-related deaths in 2023, with the majority occurring in sub-Saharan Africa [1]. *Anopheles* mosquitoes, particularly species from the *Anopheles gambiae* complex and *Anopheles funestus* group, are the primary vectors responsible for the transmission of *Plasmodium*, the parasite that causes malaria [2].

While malaria remains a significant challenge worldwide, countries like South Africa have made remarkable strides in reducing its burden. For example, malaria prevalence has been significantly reduced, with cases now primarily confined to the three north-eastern provinces bordering Botswana, the Kingdom of Eswatini, Mozambique and Zimbabwe [3, 4]. In this region, most transmissions are classified as residual and are attributed to the *Plasmodium falciparum* parasite [4]. These transmissions are linked to five *Anopheles* species, namely *An.s funestus*, *Anopheles arabiensis*, *Anopheles merus*, *Anopheles vaneedeni* and *Anopheles parensis* [5–8]. Of these, the first two are primary vectors, while the latter three are secondary vectors. However, *An. funestus* is no longer a major threat after the re-introduction of DDT as an insecticide of choice for indoor residual spraying (IRS) in the 2000 s, which played a crucial role in the near elimination of this species [9, 10].

Past, persistent successes in vector control, notably through IRS of insecticides in houses, have emboldened South Africa to outline an ambitious malaria elimination plan [10]. This strategic initiative aims to eliminate locally acquired malaria cases by 2028, building upon the original target of 2023 [4]. A crucial aspect of achieving this goal is continuous mosquito vector surveillance to understand the entomological drivers of malaria transmission [11–14]. Indeed, surveillance is key to malaria elimination and is one of the three pillars for malaria control and elimination [13]. Vector surveillance involves collecting key entomological indicators that guide the implementation of appropriate malaria control methods and products. It focuses on monitoring vector population dynamics, assesses malaria transmission risk including receptivity, guides targeted interventions and evaluates the effectiveness of control programmes [12–14]. By offering insights into transmission patterns and the effectiveness of vector control measures, surveillance enables public health authorities to tailor strategies to local conditions and emerging threats, ultimately supporting efforts to reduce and eliminate malaria. This requires the use of a variety of malaria vector-sampling tools.

Actually, South Africa’s malaria control programmes (MCPs) rely on a range of techniques for vector surveillance. These include larval sampling in potential breeding sites, intermittent house searches and pyrethrum spray catches (PSC) which are only done in response to malaria cases being recorded in an area [11]. However, these methods are not sufficiently robust for routine monitoring of malaria vector populations at low densities (i.e. low transmission settings). Larval collections, for instance, are resource-intensive and provide qualitative rather than quantitative abundance measures, a pivotal determinant of transmission intensity [15–17]. House searches demand intrusive efforts and necessitate the presence of at least one household member, often hindered by work commitments. Despite their efficiency, pyrethrum spray catches are compromised by insecticide resistance and operational challenges [18]. Considering these limitations, it becomes imperative for the MCPs to adapt or add alternative adult mosquito collection methods to their vector surveillance tool kit.

There is a range of adult mosquito surveillance tools, each offering varying degrees of sensitivity and specificity to the collection of entomological data. These include human landing catches (HLC), Centres for Disease Control light traps (CDC-LT), odour-baited traps, window exit traps, containers, and others [19]. However, the suitability of each of these tools largely depends on the MCPs vector surveillance objectives [20]. In settings with low mosquito population densities for example, methods such as clay pot deployments are an effective mosquito sampling tool [8]. This method capitalizes on the natural resting behaviour of anopheline mosquitoes, making it a promising tool for capturing both indoor- and outdoor-resting mosquitoes [8]. Odour-baited traps such as CO_2_-baited tents mimic human chemical cues that attract host-seeking mosquitoes, aiding in the collection of mosquitoes in search of a blood meal [21–25]. Lastly, HLC provides invaluable insights into the behaviour and abundance of mosquitoes that actively seek out human hosts [18–20, 26]. All these techniques offer unique advantages suited to specific surveillance goals. There is, therefore, a need to choose the right set of sampling techniques applicable under a low malaria transmission setting i.e. a set of techniques capable of collecting mosquitoes in low-density settings while providing a suitable range of entomological indicators.

Given the limitations of current surveillance methods and the need for more effective monitoring in low transmission settings, this study aimed to compare alternative vector surveillance tools against standard methods. Specifically, it compared the suitability of three collection methods in terms of species composition, relative abundance, richness, and diversity to determine their effectiveness for routine surveillance in such settings. Additionally, the study investigated whether clay pots can provide sufficient samples to gather essential entomological indicators needed to monitor vector populations in areas with low malaria transmission.

## Methods

### Field study site

**Fig. 1:**
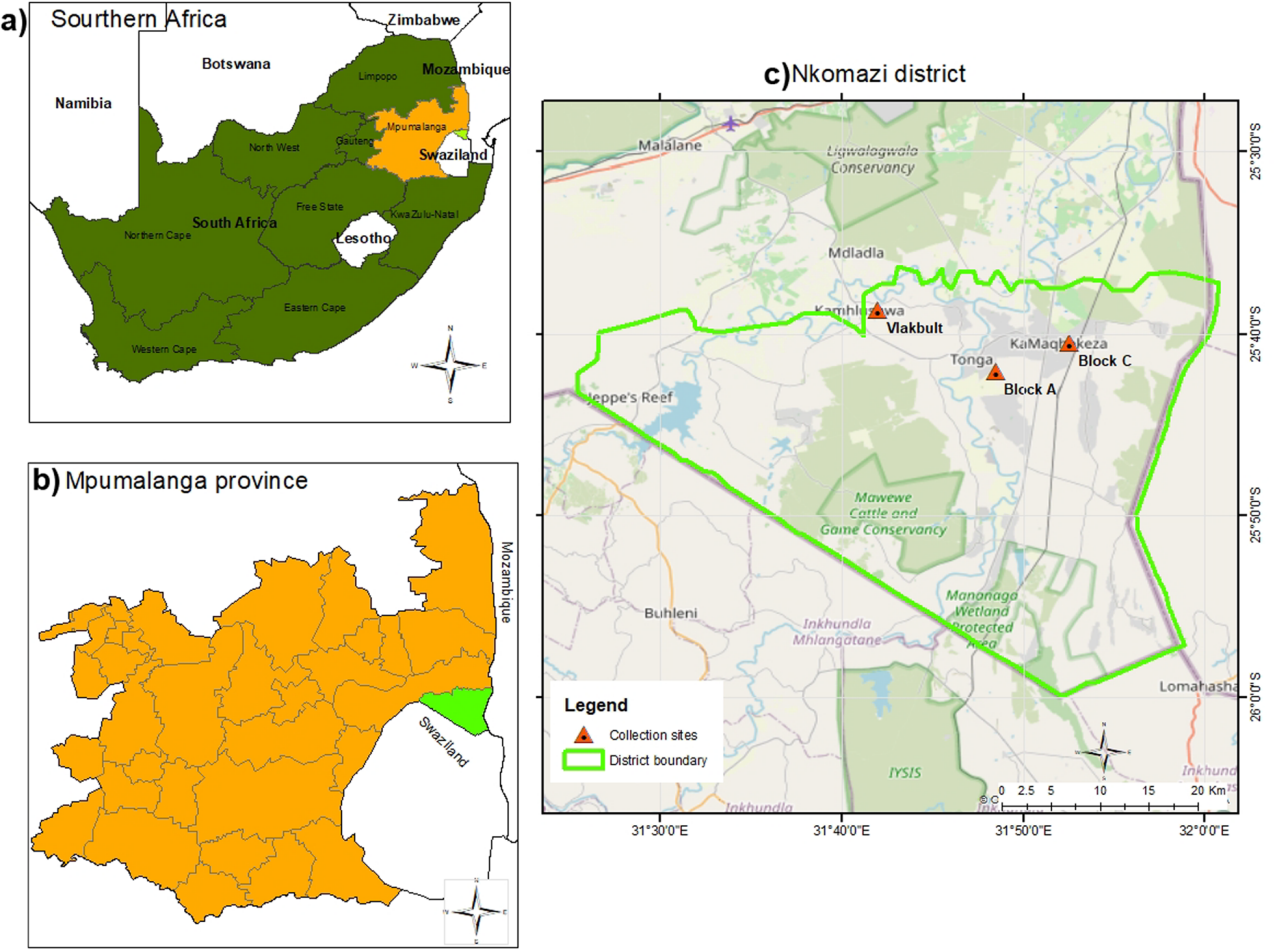
An ArcMap version 10.8.2 generated map depicting the study site: **a** a map of Southern Africa showing South Africa and its neighbouring countries (top left), **b** Mpumalanga Province (bottom left), **c** Nkomazi local municipality (right), with Block A, Block C and Vlakbult collection sites indicated by red triangles

This study was done in the Nkomazi local municipality, situated in the Ehlanzeni District Municipality of Mpumalanga Province, South Africa (Fig. 1) from March 2019 to March 2020. The municipality is categorized as a malaria high-risk region, with an approximate incidence rate of 500 cases per 100 000 inhabitants [27]. Geographically, the municipality shares borders with Mozambique to the east, and Eswatini to the south, making it receptive to malaria parasites seeding from these high malaria burden countries. Economic activities in the region include intensive sugarcane cultivation, as well as fruit and vegetable farming under irrigation, which creates potential anopheline breeding sites. Climatically, the dry season spans from June to September, while the rainy season spans from November to May. The average annual rainfall ranges between 1200 and 1800 mm, with mean daily temperatures fluctuating between 25 °C and 35 °C. Relative humidity averages between 65 and 80%.

The area features a mix of traditional thatched houses and modern iron-roofed structures, constructed using materials like bricks or mud. Three sampling sites within the Nkomazi local municipality were selected based on the following criteria: (1) a history of malaria cases or a high malaria incidence, (2) a substantial abundance of *Anopheles* mosquitoes, and (3) ecological similarities. These were Block A (S25°42′03″; E31°48′31″), Block C (S25°40′30″; E31°52′37″), and Vlakbult (S25°38′42″; E31°42′01″). The distances between the sites are as follows: Block A to Block C = 7.43 km, Block A to Vlakbult = 12.51 km, and Block C to Vlakbult = 18.02 km. All three sites share the same climate, malaria epidemiology, and ecological characteristics [28].

### Mosquito collection methods

**Fig. 2:**
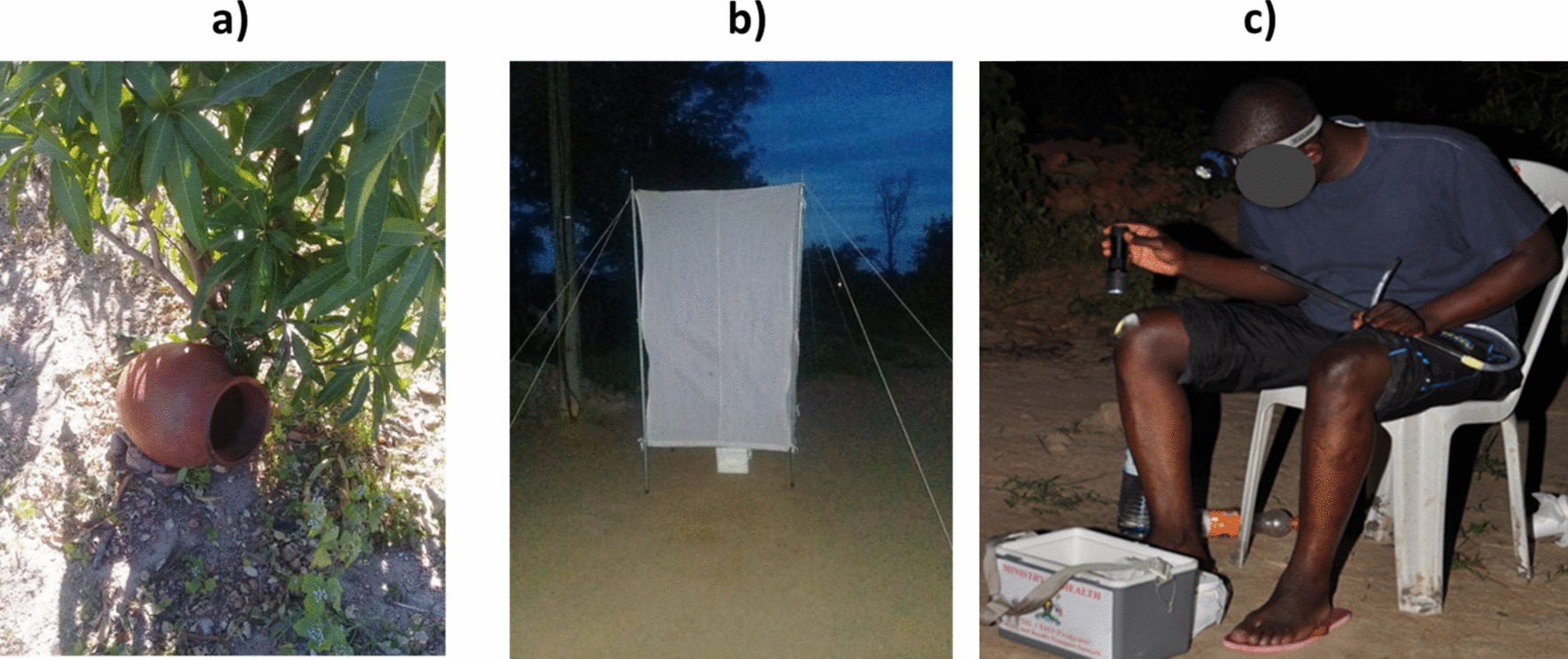
Collection methods tested during this study: **a** clay pot, **b** CO_2_-baited tent, and **c** human landing catch (HLC)

Three distinct mosquito collection methods i.e. clay pots, CO_2_-baited tents and HLC, were utilized in this study (Fig. 2). These were chosen after preliminary surveillance using other traditional sampling methods that presented challenges, leading to their exclusion/discontinuation from this study (Appendix 1, Fig. S1).

Clay pots (Fig. 2a) were similar to those used in other studies [6, 8] and were locally sourced and had capacities ranging from 20 to 25 L. Each pot had an opening of approximately 20 cm in diameter. The pots were strategically placed on the ground at a 45° angle in a shaded location and were oriented towards the north or south to minimize exposure to direct sunlight.

The CO_2_-baited tent was constructed using four stainless steel rods covered with a fine-meshed nylon net. The net featured a zipped door measuring 100 cm in length to facilitate entry and exit of collectors. A gap of 10–30 cm was deliberately left between the bottom of the tent and the ground to allow mosquitoes to enter. The structural integrity of the tent was maintained by tension ropes, ensuring stability during data collection. To attract mosquitoes, dry ice pellets, totalling at least 800 g, were placed inside an insulated container filled with sawdust and positioned centrally within the tent.

HLC were conducted by trained and experienced mosquito collectors aged over 18, who sat on a chair and exposed their lower limbs, from knee to ankle, to serve as bait for host-seeking mosquitoes (Fig. 2c).

### Study design and Anopheles mosquito sampling

Households were first stratified by history of having recorded a malaria case. Subsequently, from the households that had recent cases, one household each from Block A, Block C and Vlakbult was randomly selected. Verbal informed consent to participate was obtained from all participating households before study activities commenced. Each of the three collection methods, namely clay pots, CO_2_-baited tents and HLC, were strategically positioned outdoors at fixed sampling points within the index household of each site.

Host-seeking mosquitoes were collected hourly from 06:00 pm to 06:00 am using both HLC and CO_2_-baited tents. For HLC, two collectors per collection site, seated at least 1 m apart and at least 50 m from other collection methods, were used. These collectors, equipped with torches and aspirators, collected mosquitoes as they attempted to feed on exposed lower limbs. Additionally, they took 5–10-min breaks each hour to collect mosquitoes from the CO_2_-baited tent.

The same collectors were responsible for collecting mosquitoes resting in clay pots in the morning between 06:00 am and 08:00 am. Each collector spent approximately 5–10 min to clear the mosquitoes from each clay pot. The same sampling locations were maintained throughout the week, with rotations and shift swaps among the teams to mitigate bias in attractiveness between individuals.

Collections occurred for five consecutive nights and mornings for clay pots and after every 4–6 weeks. The sampling started from March 2019 to March 2020. The specific periods included March, April, May, November, and December 2019, as well as February and March 2020. In total, this constituted seven collection time points. Collection activities were not done during the winter months of June, July, and August, as well as in January 2020, due to resource constraints. Seasons were defined as spring: (September, October, and November); summer: (December, January, and February); autumn: (March, April, and May) and winter: (June, July, and August).

### Meteorological variables

All meteorological variables were obtained from the South African Weather Service [29] through its Komatidraai Weather Station records (S25°30′50″; E31°54′46″). These variables included air temperature (°C), relative humidity (%), daily rainfall (mm) and wind speed (m/s), all of which play crucial roles in influencing mosquito behaviour, population dynamics and malaria transmission (Fig. S2).

### Sample processing

#### Field processing of mosquito specimens

Following each collection day, mosquitoes were carefully transferred into individual 100 millilitres paper coffee cups covered with a fine meshed net secured by an elastic rubber band. Each cup was appropriately labelled to indicate the collection site, collection method and collection date. A 10% sugar solution, soaked into a cotton ball, was provided to each cup to sustain the collected mosquitoes.

Any dead mosquitoes were placed in separate 1.5 millilitres Eppendorf tubes containing silica gel separated by a blank paper for preservation. Subsequently, all mosquitoes were transported to the National Institute for Communicable Diseases (NICD) in Johannesburg, South Africa, for further laboratory processing of samples.

#### Laboratory analysis of mosquito specimens

All collected mosquitoes were labelled with their own specific identification sticker and subjected to morphological identification using dichotomous taxonomic identification keys [30, 31]. *Anopheles* specimens morphologically identified as *An. gambiae* complex or *An. funestus* group underwent further identification to species by PCR [32, 33]. The molecular PCR assays for the *An. gambiae* complex distinguish between *An. merus*/*melas*, *An. arabiensis*, *An. gambiae* and *An. quadriannulatus*. The *An. funestus* group PCR assay differentiates between *An. funestus*, *An. vaneedeni*, *Anopheles rivulorum*, *Anopheles rivulorum*-like, *An. parensis*, and *Anopheles leesoni*. No further molecular identification was necessary for the samples of *Anopheles coustani*, *Anopheles demeilloni*, *Anopheles maculipalpis*, *Anopheles marshalii*, *Anopheles pharoensis*, *Anopheles pretoriensis*, *Anopheles rufipes*, *Anopheles squamosus*, *Anopheles tenebrosus* and *Anopheles ziemanni*.

### Statistical analysis

All data were captured into a Microsoft Excel file, version 2016 (Microsoft Corporation, Redmond, WA). All statistical analysis and data visualization were carried out using the R software, version 4.3.1. [34]. The statistical significance was set at p < 0.05 for all analyses.

Species composition was summarized as percentages against total collected. Relative abundance between different collection sites and productivity of each sampling method was analysed using a Kruskal–Wallis test due to data abnormality and non-homogenous distribution. Species that accounted for less than 0.5% of total abundance were not included in the statistical analysis. Species richness and Shannon diversity index for each collection site and method were compared using ANOVA and Tukey’s *posthoc* test where significant differences were found.

The association/effect of the different collection methods on species abundance considering other factors such as collection site, year, season, and sex as fixed effects were evaluated using a Generalized Linear Mixed Model (GLMM) with Negative Binomial Regression distribution. Meteorological variables (temperature, relative humidity, rainfall, and wind speed) were excluded from the model as they remained constant across all collection sites. Model fit statistics (Aikaike Information Criterion (AIC), Bayesian information criterion (BIC) and log-likelihood) were used to select the best model. A positive coefficient estimate indicated a higher species capture rate compared to the reference method (HLC).

## Results

### Anopheles species composition

A total of 1337 *Anopheles* mosquitoes was collected throughout this study, composed in predominance of females (98.5%, n = 1317) compared to males (1.5%. n = 20) (Table 1). The highest collections were from Block A (59.5%, n = 796) followed by Vlakbult (26.8%, n = 358) and Block C (13.7%, n = 183). In terms of collection method, CO_2_-baited tents collected the most specimens (57.6%, n = 770), followed by HLC (39.1%, n = 523) and clay pots (3.3%, n = 44). Most collections occurred in autumn (49.8%, n = 666), followed by spring (26.7%, n = 357), and summer (23.5%, n = 314).

**Table 1:** *Anopheles* mosquitoes sampled between March 2019 and March 2020 in Nkomazi local municipality, Ehlanzeni district, Mpumalanga, South Africa, stratified by sex, collection site, collection method, season, and species

Variable			Total (% of total collected) (%)
Sex	Female		1317 (98.5)
	Male		20 (1.5)
Collection site	Block A		796 (59.5)
	Block C		183 (13.7)
	Vlakbult		358 (26.8)
Collection method	Clay pot		44 (3.3)
	CO_2_-baited tent		770 (57.6)
	HLC		523 (39.1)
Season	Autumn		666 (49.8)
	Spring		357 (26.7)
	Summer		314 (23.5)
Species collected	*An. gambiae complex*	*An. arabiensis*	131 (9.8)
		*An. merus*	504 (37.7)
		*An. quadriannulatus*	11 (0.8)
		An. gambiae complex*	54 (4.0)
		Total	700 (52.4)
	*An. funestus* group	*An. leesoni*	3 (0.2)
		*An. rivulorum*	125 (9.4)
		*An. vaneedeni*	15 (1.1)
		*An. funestus* group*	39 (2.9)
		Total	182 (13.6)
	*Other Anophelines*	*An. coustani*	88 (6.6)
		*An. demeilloni*	4 (0.3)
		*An. maculipalpis*	212 (15.9)
		*An. marshalii*	16 (1.2)
		*An. pharoensis*	6 (0.4)
		*An. pretoriensis*	4 (0.3)
		*An. rufipes*	97 (7.3)
		*An. squamosus*	21 (1.6)
		*An. tenebrosus*	4 (0.3)
		*An. ziemmanni*	3 (0.2)
		Total	455 (34.0)
Total			1337 (100)

^*^Indicates specimens that could not be identified to species by PCR (i.e., did not amplify on a gel)

All collected *Anopheles* mosquitoes were identified and classified to genus level based on morphological features. Among these were members of the *An. gambiae* complex (52.4%, n = 700) and the *An. funestus* group (13.6%, n = 182). Further molecular analysis of the *An. gambiae* complex revealed their distribution as: *An. merus* (72.0%, n = 504), *An. arabiensis* (18.7%, n = 131), *An. quadriannulatus* (1.6%, n = 11) as well as those that could not be identified by PCR (7.7%, n = 54). For the *An. funestus* group, molecular identification indicated that *An. rivulorum* contributed the majority at 68.7% (n = 125), followed by *An. vaneedeni* (8.2%, n = 15), *An. leesoni* (1.7%, n = 3) and other unidentified specimens (21.4%, n = 39). Additionally, various other anophelines were identified, including *An. maculipalpis* (15.9%, n = 212), *An. rufipes* (7.3%, n = 97), *An. coustani* (6.6%, n = 88), *An. squamosus* (1.6%, n = 21), *An. marshalii* (1.2%, n = 16), *An. pharoensis* (0.4%, n = 6), *An. demeilloni* (0.3%, n = 4), *An. pretoriensis* (0.3%, n = 4), *An. tenebrosus* (0.3%, n = 4), and *An. ziemanni* (0.2%, n = 3).

### Relative abundance of collected Anopheles mosquitoes

#### Relative abundance of Anopheles mosquitoes by collection site

Relative abundance based on collecting site showed no significant difference (Kruskal–Wallis: χ^2^ = 4.89, df = 2, p = 0. 087). The dominating species in Block A was *An. merus*, representing 58.2% (n = 463) of the total number of mosquitoes collected from this block, followed by *An. arabiensis* (12.2%, n = 97) and lastly *An. maculipalpis* (11.4%, n = 91). In Block C, *An. rivulorum* was the dominant species, constituting 25.7% (n = 47) of the total collections from this area, followed by *An. coustani* (21.3%, n = 39) and *An. merus* (19.7%, n = 36). The predominant species at Vlakbult was *An. maculipalpis*, accounting for 33.8% (n = 121) of the total number, followed by *An. rufipes* (18.4%, n = 66) and *An. rivulorum* (15.6%, n = 56).

#### Relative abundance of Anopheles mosquitoes by collection method

The relative abundance of *Anopheles* mosquitoes by collection method also showed no significant variation (Kruskal–Wallis: χ^2^ = 5.4158, df = 2, p = 0.067). *Anopheles maculipalpis* and *An. merus* were the main species collected by CO_2_-baited tent trap with relative abundance of 27.3% (n = 210) and 23.9% (n = 184), respectively. Other significant species collected by CO_2_ tent trap include *An. rivulorum* (11.7%, n = 90) and *An. rufipes* (11.2%, n = 86). For clay pots, *An. arabiensis* and *An. merus* were the predominant species, with relative abundances of 43.2% (n = 19) and 29.5% (n = 13) respectively. The predominant species from HLC were *An. merus* with relative abundance of 58.7% (n = 307) followed by *An. arabiensis* with relative abundance of 15.9% (n = 83). Another notable species collected using HLC was *An. vaneedeni* with a relative abundance of 1.3% (n = 7).

### Species richness and diversity

#### Species richness and diversity by collection site

**Fig. 3:**
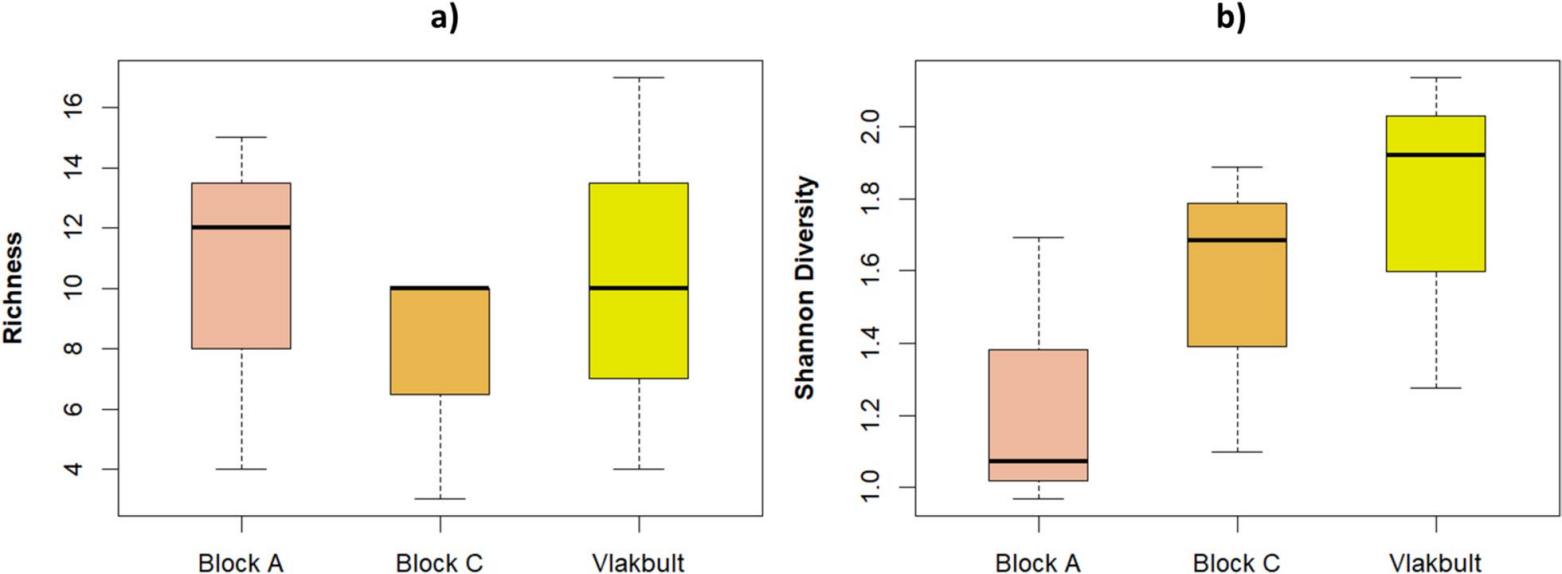
*Anopheles* species distribution index: **a** richness and **b** Shannon diversity per collection site in Ehlanzeni District Municipality, Mpumalanga Province, South Africa, 2019–2020

The number of species collected varied across the different sites, with both Block A and Vlakbult yielding 17 species, while Block C had 12 species. However, species richness did not show significant differences between the sites (mean ± SD: Block A: 10.3 ± 5.7, Block C: 7.67 ± 4.0, Vlakbult: 10.3 ± 6.5; ANOVA: F _(2,6)_ = 0.23, p = 0.8) (Fig. 3a). Likewise, Shannon diversity showed no significant variation across the sites (mean ± SD: Block A: 1.24 ± 0.4, Block C: 1.56 ± 0.4, Vlakbult: 1.78 ± 0.4; F _(2,6)_ = 1.24, p = 0.36) (Fig. 3b).

#### Species richness and diversity by collection method

**Fig. 4:**
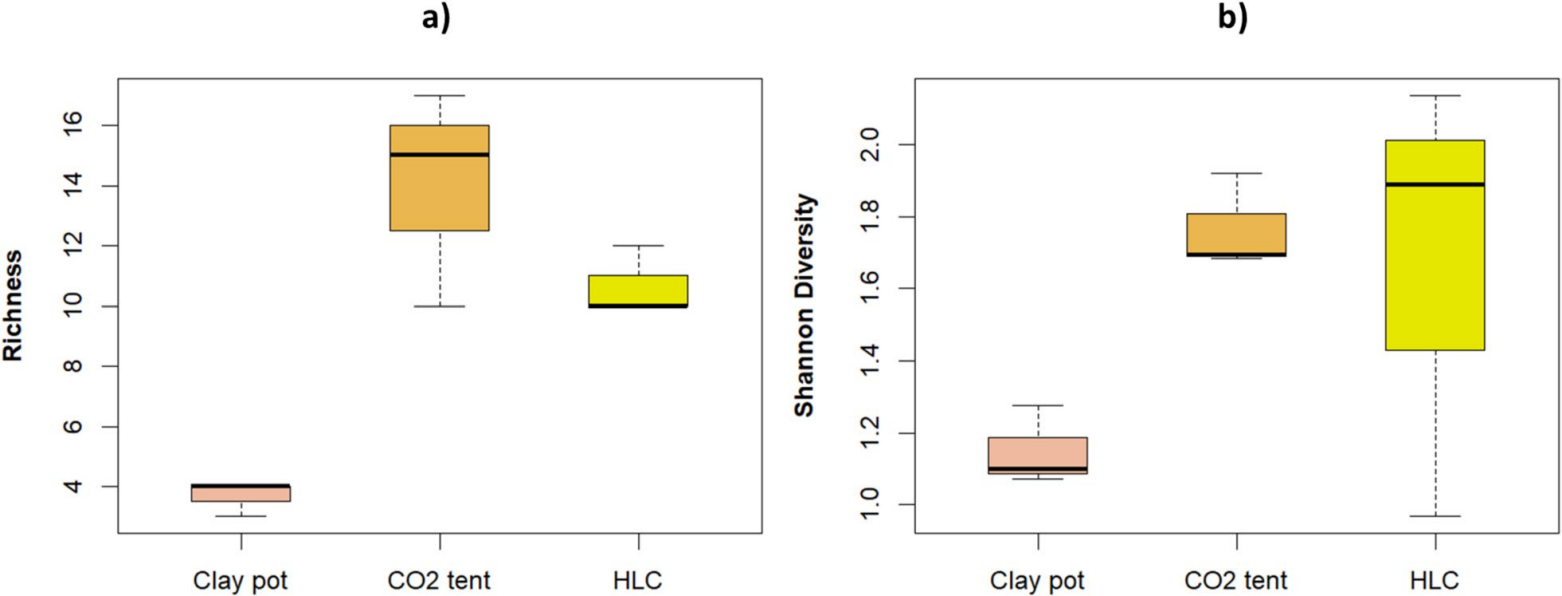
*Anopheles* species distribution index: **a** richness and **b** Shannon diversity per collection method in Ehlanzeni District Municipality, Mpumalanga Province, South Africa, 2019–2020

In terms of collection methods, the number of species captured varied significantly, with 17 species collected using CO_2_-baited tents, 14 species with HLC, and 7 species with clay pots. There were significant differences in species richness among the collection methods (mean ± SD: clay pot: 3.7 ± 0.6, CO_2_-baited tent: 14 ± 3.6, HLC: 10.7 ± 1.2; ANOVA: F _(2,6)_ = 17.07, p = 0.003), with the CO_2_-baited tent showing significantly greater richness compared to the clay pot (p = 0.003) and HLC (p = 0.019) (Fig. 4a). However, no significant differences were observed in Shannon diversity across the methods (mean ± SD: clay pot: 1.6 ± 0.1, CO_2_-baited tent: 1.7 ± 0.1, HLC: 1.6 ± 0.6; ANOVA: F _(2,6)_ = 2.4, p = 0.17) (Fig. 4b).

#### Overall association/effect of collection methods to species count, considering collection site, year, season, and sex

The results of the negative binomial regression model which was utilized to assess the impact of various factors on species count are summarized in Table 2. The model included collection method, collection site, month, and sex as predictors. HLC was the point of reference against which the other collection methods were compared. Clay pots were associated with a significant decrease in species count (Estimate = − 0.7293, p = 0.027) whilst CO_2_-baited tents showed an increase in species count, but this effect is not statistically significant (Estimate = 0.2085, p = 0.222). Compared to the reference month (April), February and November were associated with significant increases in species count (Estimates = 0.6724 and 0.7708; p = 0.017 and 0.007, respectively). May showed a marginally significant decrease in species count (Estimate = − 0.6145, p = 0.051). December and March did not show significant effects. Male specimens were associated with a significant decrease in species count compared to females (Estimate = − 1.3137, p = 0.002). Compared to Block A, both Block C and Vlakbult were associated with significant decreases in species count (Estimates = − 0.8206 and − 0.7498; p < 0.001 for both).

**Table 2:** The results of the negative binomial regression model which was utilised to assess the impact of various factors on species count

Predictor	Estimate	Std. Error	z. value	Pr (>|z|)
Intercept	1.8353	0.2340	7.842	4.44e-15***
Clay pot	− 0.7293	0.3295	− 2.213	0.02687*
CO_2_-baited tent	0.2085	0.1706	1.222	0.22156
Block C	− 0.8206	0.2079	− 3.947	7.93e-05***
Vlakbult	− 0.7498	0.1853	− 4.047	5.19e-05***
December	− 0.2851	0.3410	− 0.836	0.40306
February	0.6724	0.2804	2.398	0.01650*
March	0.2117	0.2411	0.878	0.37994
May	− 0.6145	0.3144	− 1.955	0.05060^
November	0.7708	0.2880	2.676	0.00745**
Male	− 1.3137	0.4164	− 3.155	0.00161**

Significance codes: ***: p ≤ 0.0001, **: p ≤ 0.001, *: p ≤ 0.01, ^: p ≤ 0.05, (blank): p > 0.1

## Discussion

A range of *Anopheles* mosquitoes was collected during this study, including those from the *An. gambiae* complex and *An. funestus* group, which have been previously identified as malaria vectors in South Africa [30, 35]. Additionally, several other *Anopheles* species, known to play a role in malaria transmission in various regions across Africa, were also collected.

From the *An. gambiae* complex, only *An. arabiensis*, *An. merus*, and *An. quadriannulatus* were collected. This aligns with previous studies that have noted the presence of these species in South Africa [8–10, 36–40]. *Anopheles arabiensis* is recognized as a primary vector of residual malaria in South Africa [8, 40]. Its variable feeding and resting behaviours make it less susceptible to IRS control measures [41–43]. Although *An. merus* is considered a potential secondary vector, there is no direct evidence linking it to malaria transmission in South Africa [39], but it has been confirmed as a vector in other regions including Tanzania and neighbouring Mozambique [44–47]. Its presence in Mpumalanga Province, where it was predominantly captured using HLC, suggests its possible role in malaria transmission [36, 38, 39]. In contrast, *An. quadriannulatus*, although common in southern and eastern Africa, is primarily considered a non-vector due to its zoophilic behaviour [48–56].

From the *An. funestus* group, *An. vaneedeni* (from the *An. funestus* sub-group), *An. leesoni* (from the *An. minimus* sub-group) and *An. rivulorum* (from the *An. rivulorum* sub-group) were identified. These species have also been reported in previous studies conducted in South Africa [6, 8, 40, 56, 57]. While *An. leesoni* has not been confirmed as a malaria vector in South Africa, though it has been recognized as a potential minor vector in Tanzania [58], Cameroon [59], and Nigeria [60]. *Anopheles rivulorum* has also not been implicated as a malaria vector in South Africa although it is a known secondary vector in regions like Kenya [61], Tanzania [58], and Zambia [53]. *Anopheles vaneedeni*, however, is the only species in the *An. funestus* group identified as a secondary vector in South Africa based on field samples testing positive for *P. falciparum* [6].

It is important to note that some species identified morphologically as members of the *An. gambiae* complex or *An. funestus* group did not successfully amplify during molecular identification (7.7% for the *An. gambiae* complex and 21.4% for the *An. funestus* group). This may be due to misidentification by experienced entomologists using morphological keys, especially when specimens had damaged or indistinct features [30, 62]. Misidentification can have significant implications for vector control efforts, as demonstrated by a misidentification incident in Zimbabwe during the 1970 s that led to ineffective insecticide use [63, 64]. Further species identification of collected samples to species revealed insecticide resistance in *An. arabiensis* and susceptibility in *An. quadriannulatus*, prompting a revision of IRS strategies. Additionally, misidentification between species within the *An. gambiae* complex and *An. leesoni* highlights the need for enhanced accuracy in species identification [62]. To minimize misidentification, integrating molecular techniques alongside morphological identification methods is critical, as demonstrated in this study. Institutions with sufficient resources may also consider advanced molecular techniques such as next-generation sequencing or sequencing of the ribosomal DNA regions, to better understand genetic diversity and population structure within malaria vector populations [65, 66].

Other *Anopheles* species collected in this study (*An. coustani, An. demeilloni, An. maculipalpis, An. marshalii, An. pharoensis, An. pretoriensis, An. rufipes, An. squamosus, An. tenebrosus*, and *An. ziemanni*) have been documented in various regions in South Africa and neighbouring countries [6–8, 39, 57, 67, 68]. These species have different distributions, behaviours, and roles in malaria transmission [69, 70]. For instance, *An. coustani* has been shown to carry *Plasmodium* sporozoites in multiple African countries, including the Democratic Republic of Congo [71], Kenya [72, 73], Tanzania [74] and Madagascar [75, 76], indicating its potential as a secondary vector. *Anopheles demeilloni* has been identified as a malaria vector in Ethiopia but has also been observed feeding on cattle blood [77–79]. Similarly, *An. pharoensis* is considered a secondary malaria vector and is known to feed on both human and bovine blood [80, 81]. Other species, such as *An. pretoriensis, An. rufipes, An. squamosus, An. tenebrosus*, and *An. ziemanni*, have also been identified as potential secondary vectors in different African countries [82–91]. Research into the malaria transmission potential of *An. maculipalpis* and *An. marshalii* is limited and warrants further research [30, 35, 92].

In terms of relative abundance of *Anopheles* mosquito species, this varied significantly between collection sites and collection methods. At Block A, *An. merus* was the most frequently collected species, comprising over half of the total catch, followed by *An. arabiensis* and *An. maculipalpis*. In contrast, Block C’s dominant species was *An. rivulorum*, with *An. coustani* and *An. merus* as less prevalent species. At Vlakbult, *An. maculipalpis* was the most abundant, with *An. rufipes* and *An. rivulorum* also being common. Despite these site-specific differences, statistical analyses showed no significant differences in overall *Anopheles* abundance between sites. This finding aligns with previous studies that reported variations in mosquito species relative abundance across different areas in Mpumalanga Province [38, 39]. Environmental factors, collection methods, and human activities likely influence these population dynamics, and future research should consider these variables [72, 93, 94].

In this study, collection methods influenced the species captured. CO_2_-baited tents predominantly captured *An. maculipalpis* and *An. merus*, while clay pots were more effective in sampling *An. arabiensis* and *An. merus*. HLC yielded the highest number of *An. merus*, followed by *An. arabiensis*. Despite these variations in species capture, statistical analyses revealed no significant differences in the overall relative abundance of *Anopheles* species across collection methods. This suggests that while certain methods may be more effective for capturing specific species, the overall abundance remains consistent across methods. The observed variations highlight the need to consider method-specific biases, such as differential attraction or trapping efficiency [95, 96]. Other studies have similarly reported variations in species relative abundance across different collection methods [8, 40, 82, 97]. This study’s specificity is valuable for targeted species surveillance, enabling the selection of appropriate methods for collecting particular species. Additionally, employing multiple collection methods at targeted sites can provide a comprehensive understanding of mosquito species relative abundance, thereby informing tailored vector control strategies. However, it is important to also note that the availability of resources may limit the deployment of multiple methods for routine surveillance. Although this study aimed to identify the most suitable and effective method for vector surveillance in a low malaria transmission setting, using multiple methods would yield more comprehensive and accurate data, but at a higher cost. This increased cost may be prohibitive for many national MCPs, which may not have the financial resources to implement such approaches routinely.

This study provides valuable insights into species richness and diversity across collection methods and sites. Our analysis revealed no significant differences in species richness or Shannon diversity across the different collection sites (Block A, Block C and Vlakbult), suggesting that the ecological conditions in these locations may be similar in terms of species availability or habitat quality [98–100]. The average richness observed in Block A and Vlakbult was relatively high, while Block C exhibited lower richness, though not statistically significant.

However, the collection methods showed significant differences in species richness. The CO_2_-baited tent method yielded notably higher species richness compared to clay pots and HLC, supporting findings that certain collection techniques are more effective for capturing specific taxa [95, 96, 101]. The clay pot method, while simpler, appears to underestimate species richness in this context.

Interestingly, despite the significant differences in species richness, Shannon diversity did not show comparable variation, suggesting that the distribution of individuals among species was similar across methods [102–105]. This finding highlights the importance of considering both richness and diversity when assessing ecological communities, as richness alone may not provide a complete picture of community structure [106].

Overall, the results of this study underscore the importance of both collection site and method in determining mosquito species richness and diversity. The significant differences in species richness among collection methods advocate for a more integrated approach to entomological surveillance, where multiple methods are utilized to capture the full diversity of mosquito populations. By leveraging the strengths of different collection techniques, researchers and public health practitioners can enhance their understanding of mosquito dynamics, which is vital for effective malaria vector surveillance and control strategies.

The analysis to assess the impact of various factors on species count indicated that collection method, collection site, month, and sex significantly influence species counts. Specifically, clay pots were less effective than HLC, while CO_2_-baited tents did not significantly impact species identified when compared to HLC. Species counts were lower in Block C and Vlakbult compared to Block A. Additionally, species counts varied across months, with February and November showing higher counts. Male specimens were less prevalent than females. These findings underscore the effectiveness of various collection methods, collection sites, the importance of timing collections, and the disparities in capture rates between male and female mosquitoes, which can inform future entomological surveillance strategies for malaria vector monitoring. Additionally, each collection method demonstrated distinct strengths and limitations, influenced by factors such as time of year, seasonal variations, mosquito sex, and specific collection sites.

CO_2_-baited tents proved to be the most productive in capturing a high total number of mosquitoes. This method’s reliance on CO_2_ as a lure in this study enabled it to attract a broad spectrum of host-seeking mosquitoes. However, its non-selective nature means it captures a variety of mosquitoes, not exclusively malaria vectors. This lack of selectivity highlights limits on the application of CO_2_-baited tents for species specific surveillance requirements. While they are excellent for overall species sampling, they may not be ideal for targeted collection of specific malaria vectors. The dependence on dry ice in this study, which can be challenging to source in rural or low-density areas, and the failure of alternatives such as yeast [21] in preliminary trials, underscore the need for improvements. Future developments could focus on optimizing the CO_2_ sources and release mechanisms, exploring more accessible or effective lures, or incorporating additional attractants to enhance the targeting of malaria vectors.

HLC demonstrated high effectiveness for capturing members of the *An. gambiae* complex, including *An. arabiensis* females. This is notable given that *An. arabiensis* typically feeds on animals rather than humans but is also known for its opportunistic/behavioural feeding plasticity [103]. The ability of HLCs to capture this species highlights the complex interactions between mosquitoes and their hosts and reiterates this method’s status as the gold standard for measuring human exposure to mosquito bites and assessing vector-host dynamics [18–20]. Nonetheless, the method faces significant ethical challenges due to the risk of exposing participants to infectious mosquito bites, particularly if appropriate anti-malarial chemoprophylaxis is not administered. The labour-intensive nature of HLCs and variability in individual attractiveness to mosquitoes also complicate its standardization and practical implementation. Future considerations for HLC use should address these ethical concerns and explore measures to minimize risks, potentially incorporating advanced protective protocols and improving standardization practices.

In this study, clay pots collected the least number of anophelines, a finding that deviates from previous studies where clay pots were reported to be more productive [8, 40, 107]. This discrepancy could stem from differences in the deployment of pots and or varying ecological conditions. In KwaZulu-Natal (KZN), South Africa, where use of clay pots has been successful, the number of clay pots per area is higher compared to the present study where they were sparely distributed [8, 40]. However, despite capturing fewer mosquitoes, clay pots were productive in collecting *An. arabiensis* and *An. merus* of both sexes, which is an important advantage for studying pathogen transmission dynamics and overwintering of pathogens [47, 72]. The portability of clay pots and their ability to be deployed in large numbers across various localities add to their utility [8, 40, 82, 107]. However, improvements are needed in the design and lures used with clay pots to better attract anthropophagic species. Refining these aspects could enhance their efficacy in capturing a larger number of malaria vectors. Additionally, addressing challenges such as the presence of mosquito predators like ants and spiders in clay pots is essential. Collection intervals by collectors also need addressing, and the addition of sticky tape or exit traps can help [82]. The versatility of clay pots, coupled with their ability to capture both male and female mosquitoes, suggests their potential surveillance value in local malaria control programs. Future research should focus on optimizing clay pot designs and lures, as well as investigating the effects of mosquito activity patterns, host-seeking behaviour, and environmental conditions on their effectiveness.

Overall, the choice of collection method influenced the species caught. Each method has unique strengths that can contribute to a comprehensive understanding of mosquito populations. While none of the methods tested are specific to anophelines and all capture other mosquito species, including *Culex*, the strengths and limitations of each approach highlight the importance of employing a combination of methods. Such an approach can mitigate biases and provide a more complete picture of mosquito species composition, abundance, and diversity.

Lastly, refining mosquito collection methods and addressing the factors that influence their effectiveness—such as seasonal variations, site-specific conditions, human and mosquito behaviour—are crucial for enhancing surveillance and control efforts. As the emergence of insecticide resistance [108] as well as invasive species, such as *Anopheles stephensi* [109], poses new challenges in Africa, maintaining vigilance, thorough surveillance, and adapting collection techniques will be vital in ensuring effective malaria control and monitoring.

## Limitations of the study

Despite providing valuable insights, this study had several limitations. First, the study design was not ideal for directly comparing the different collection methods. The collection methods were not rotated across sites or over time to account for potential positional effects, which could introduce bias. Ideally, rotating the methods would have minimized these positional effects and allowed for a more accurate comparison. Additionally, the collection methods investigated have different modes of action. For example, CO_2_-baited tents and HLC are designed for host-seeking mosquitoes, while clay pots are used to attract mosquitoes seeking a place to rest during the day or after taking a blood meal. This difference in behaviour likely contributed to a biased comparison between methods.

In addition, some available outdoor collection method alternatives, such as window exit traps and the lumin8 light trap (appendix 1), were not evaluated due to operational challenges, including theft and non-compliance by household owners, which hindered their reliable use. These practical constraints may have affected the completeness of the study and limited the range of collection methods assessed.

The geographic scope of the study was also limited, which may impact the generalizability of the results to other regions. Variations in environmental factors, such as local ecology, animal populations, and specific microclimatic conditions unique to each collection site, were not fully accounted for. These factors could have contributed to differences in mosquito diversity, behaviour, breeding patterns, and species composition, making it difficult to compare results across sites and methods accurately.

Another limitation of the study is that both the clay pots and HLC method were only used outdoors, whereas these methods can generally be employed both indoors and outdoors. This may have impacted the composition of mosquitoes collected, as certain species may prefer to feed indoors rather than outdoors. By not capturing mosquitoes indoors, the study may have missed species that are more commonly found in indoor environments, potentially leading to an incomplete representation of the mosquito population. Future studies should incorporate both indoor and outdoor collections using the clay pot and HLC methods to provide a more comprehensive understanding of mosquito species distribution and behaviour.

Finally, the data were collected over a relatively short period of 12 months, which may not have been sufficient to capture full seasonal variations in mosquito abundance and behaviour. A longer study period would have provided a better understanding of the seasonal dynamics of mosquito populations.

## Conclusion

Despite limitations, the study’s results provide valuable evidence on the comparative effectiveness of alternative mosquito collection methods compared to the standard HLC method. This information can serve as a foundation for future research studies and surveillance programs aimed at better understanding mosquito-borne disease transmission and implementing effective control measures.

This study showed that no single trapping method can provide a reliable estimate of all entomological indicators needed during mosquito vector surveillance. It is necessary to use multiple collection methods and to refine existing techniques for improved mosquito surveillance. Subsequent comprehensive evaluations of alternative adult mosquito collection methods are warranted to ensure accurate sampling and to generate key entomological surveillance indices, including vector density, species composition, insecticide susceptibility, host preferences, biting and resting behaviour, and infection rates.

## Supplementary Information

Additional file 1

## Data Availability

Data supporting the conclusions of this article are included within the manuscript.

## Abbreviations

AIC: Aikaike Information Criterion
BIC: Bayesian Information Criterion
CDC: Centre for Disease Control
CO_2_: Carbon Dioxide
GLMM: Generalized Linear Mixed Model
HLC: Human Landing Catches
KZN: KwaZulu-Natal
LT: Light Trap
MCP: Malaria Control Programme
NICD: National Institute for Communicable Diseases
PCR: Polymerase Chain Reaction
PSC: Pyrethrum Spray Catches
