# Supplementary material for: Assessing Anopheles species collection techniques in a low malaria transmission area: implications for vector surveillance and control
Source: Malar J. 2025 Jul 1;24:204. doi: 10.1186/s12936-025-05463-x (PMC12210503; doi:10.1186/s12936-025-05463-x)
Supplement: Supplementary file 1 — Additional file 1 [file 12936_2025_5463_MOESM1_ESM.docx]

Appendix

Preliminary surveillance

Preliminary surveillance and deployment of clay pots were conducted at each location towards the end of 2018 to verify the presence of *Anopheles* mosquitoes. Apart from the collection methods explored in this study, window exit traps and lumin8 light traps were also tested during the preliminary surveillance (refer to Figure S1). However, due to the theft of the lumin8-LT and discomfort caused by window exit traps, as well as non-compliance by some homeowners, these two collection methods were not further evaluated in the study.

Below is a description of the window exit traps and lumin8 light trap (LT).

Window exit traps: These traps were constructed using collapsible BugDorm-4M3030 Insect Rearing Cages (BugDorm-1H; MegaView, Taichung, Taiwan). The dimensions of the trap are W32.5 × D32.5 × H32.5 cm, with a net weight of 240 grams. The main material used is knitted mesh, with a nylon mesh size of 44 × 32 and an aperture of 650 µm. The entire cage, except for the base, is covered with mesh panels, with one opening front sleeve. To adapt the trap for mosquito collection, one side of the cage adjacent to the front sleeve was modified into a conical shape, resembling conical fishing nets. This modified section was then sealed back in an inverted manner, with the larger opening facing outward. This allowed mosquitoes escaping from inside the house through the window exit trap to be trapped and collected.

lumin8-LT: This trap is a locally produced device designed for scientific research or homestead mosquito trapping purposes (lumin8 catch ‘em; Germiston, South Africa). It has dimensions of W255 × L260 × H210 cm and is powered by an efficient 12-Watt virtually indestructible polycrystalline solar panel. The trap features multiple modes of operation, including a programmable 3 cluster variable LED variable light source with specific wavelengths for selective mosquito capture. It also offers variable suction power, a specimen net container, integrated selection mesh, and is solar-powered with a lithium-ion high-capacity battery pack.

**(a) (b) (c)**

**(d) (e)**


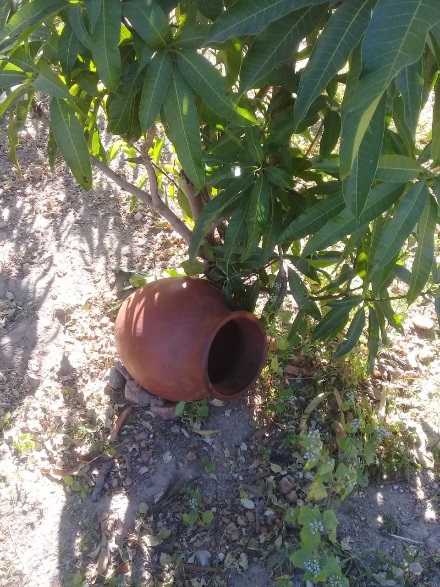

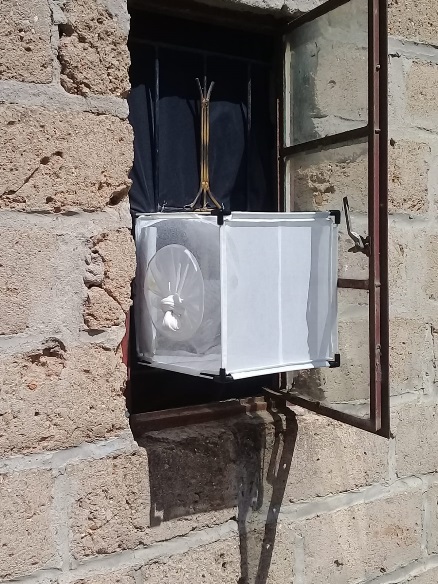

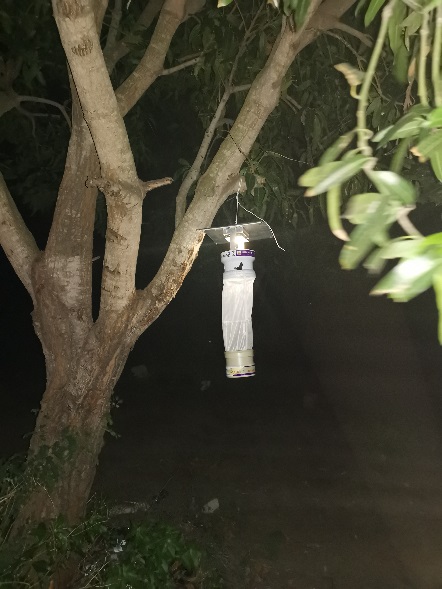

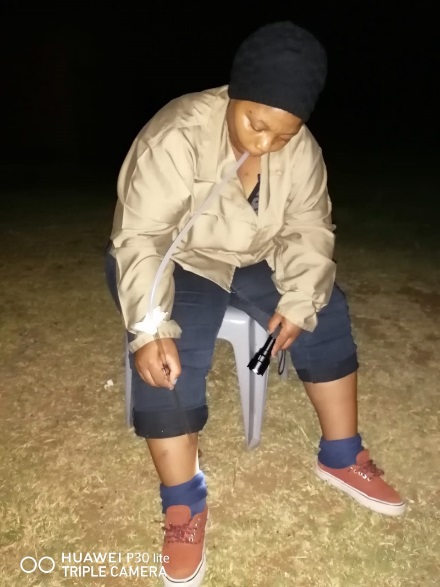

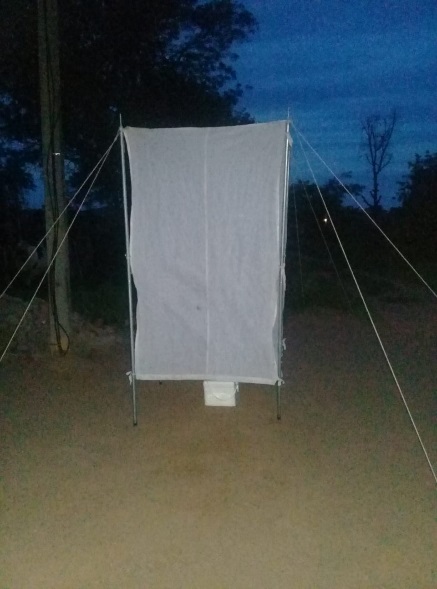


**Figure S1:** All collection methods tested during the preliminary surveillance a) Clay pots, b) Window exit traps, (c) lumin8 light trap, (d) human landing catch (HLC) and (e) CO_2_-baited tent.

Meteorological variables corresponding to the study’s collection period.


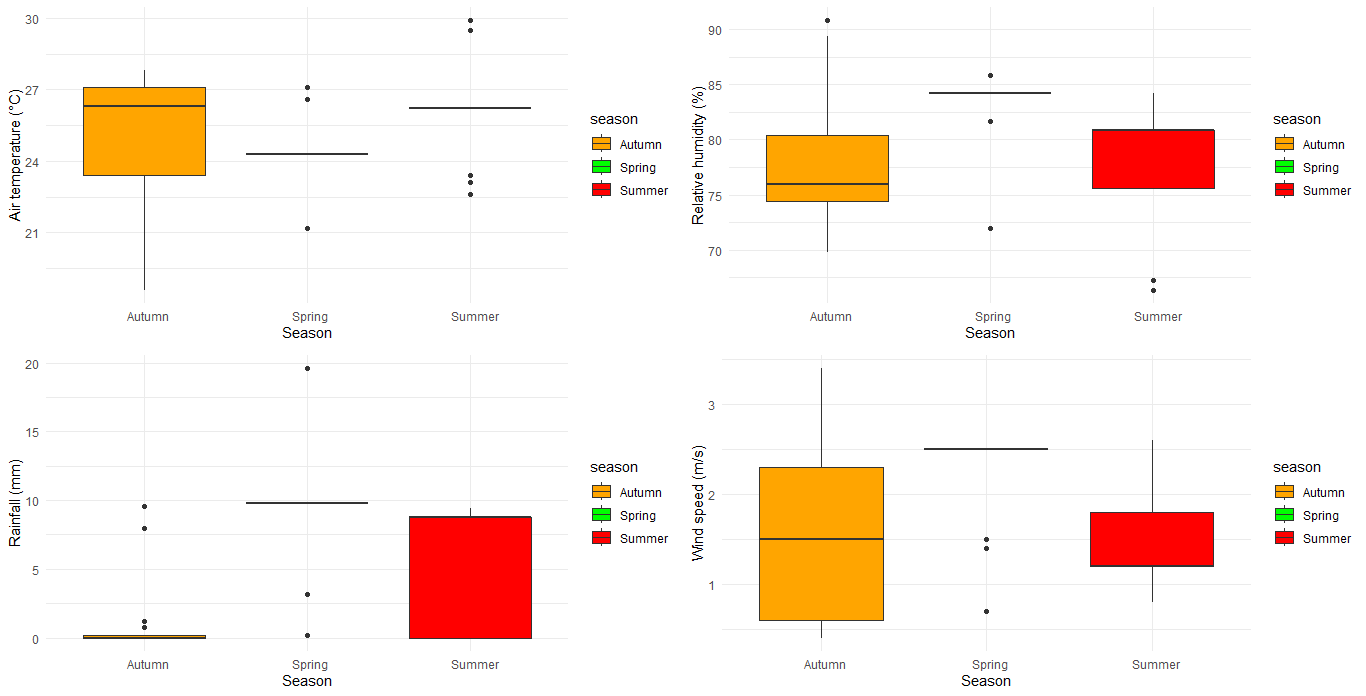


**Figure S2:** Meteorological variables (temperature (°C), relative humidity (%), rainfall (mm) and wind speed (ms−1)) corresponding to the study’s collection period/days.
